# Retinal Organoids derived from hiPSCs of an AIPL1-LCA Patient Maintain Cytoarchitecture despite Reduced levels of Mutant AIPL1

**DOI:** 10.1038/s41598-020-62047-2

**Published:** 2020-03-25

**Authors:** Dunja Lukovic, Ana Artero Castro, Koray Dogan Kaya, Daniella Munezero, Linn Gieser, Carlota Davó-Martínez, Marta Corton, Nicolás Cuenca, Anand Swaroop, Visvanathan Ramamurthy, Carmen Ayuso, Slaven Erceg

**Affiliations:** 1 Retinal Degeneration Lab, Research Center Principe Felipe, c/ Eduardo Primo Yúfera 3, 46012 Valencia, Spain; 2 National Stem Cell Bank-Valencia Node, Proteomics, Genotyping and Cell Line Platform, PRB3, Research Center Principe Felipe, c/ Eduardo Primo Yúfera 3, 46012 Valencia, Spain; 3 Neurobiology-Neurodegeneration and Repair Laboratory, National Eye Institute, National Institutes of Health, Bethesda, MD 20892 USA; 4 Departments of Ophthalmology, Biochemistry and Pharmaceutical and Pharmacological Sciences, West Virginia University, Morgantown, WV 26506 USA; 5 Department of Genetics and Genomics, IIS-Fundación Jiménez Díaz (IIS-FJD, UAM), Madrid 28040, Spain; Center for Biomedical Network Research on Rare Diseases (CIBERER), ISCIII, Madrid, Spain; 6 Department of Physiology, Genetics and Microbiology, University of Alicante, Alicante, Spain; 7 Stem Cells Therapies in Neurodegenerative Diseases Lab, Research Centre Principe Felipe, c/Eduardo Primo Yúfera 3, Valencia, Spain

**Keywords:** Induced pluripotent stem cells, Stem-cell differentiation

## Abstract

Aryl hydrocarbon receptor-interacting protein-like 1 (AIPL1) is a photoreceptor-specific chaperone that stabilizes the effector enzyme of phototransduction, cGMP phosphodiesterase 6 (PDE6). Mutations in the *AIPL1* gene cause a severe inherited retinal dystrophy, Leber congenital amaurosis type 4 (LCA4), that manifests as the loss of vision during the first year of life. In this study, we generated three-dimensional (3D) retinal organoids (ROs) from human induced pluripotent stem cells (hiPSCs) derived from an LCA4 patient carrying a Cys89Arg mutation in *AIPL1*. This study aimed to (i) explore whether the patient hiPSC-derived ROs recapitulate LCA4 disease phenotype, and (ii) generate a clinically relevant resource to investigate the molecular mechanism of disease and safely test novel therapies for LCA4 *in vitro*. We demonstrate reduced levels of the mutant AIPL1 and PDE6 proteins in patient organoids, corroborating the findings in animal models; however, patient-derived organoids maintained retinal cell cytoarchitecture despite significantly reduced levels of AIPL1.

## Introduction

Hereditary retinal degenerations are clinically and genetically heterogeneous and constitute a major cause of incurable visual impairment in working age adults. Unfortunately, this group of diseases currently lacks effective treatment options. Among the divergent clinical phenotypes, Leber congenital amaurosis (LCA) accounts for ∼5% of all inherited retinopathies and is among the most severe, with patients exhibiting visual dysfunction and losing electroretinogram signals during the early years of age^1^. Patients typically present nystagmus (repetitive, uncontrolled movements of the eyes), poor pupillary light response, and fundus abnormalities^2^. Currently, as many as 25 genes have been identified as causing LCA (https://sph.uth.edu/retnet/) primarily in an autosomal recessive manner; however, genetic defects have not been identified in almost 30% of LCA patients^2^. All known causative genes are expressed in photoreceptors and/or the retinal pigment epithelium (RPE) and associated with a wide range of functions, including phototransduction, retinoid cycling, protein trafficking, and ciliary transport.

Mutations in the aryl hydrocarbon receptor-interacting protein-like 1 (*AIPL1*) gene lead to early onset retinal disease and account for up to 5–10% of all mutations causing LCA^3^ resulting in a clinically severe form, LCA type 4 (LCA4, OMIM #604393)^4^. The *AIPL1* gene locates to chromosome 17, with 79 mutations identified to date as causing LCA (Human Genome Mutation Database). *AIPL1* encodes a 384 amino acid protein expressed only in photoreceptors and the pineal gland^5^. The protein has an FK506-binding protein (FKBP)-like domain within the N terminus, a tetratricopeptide (TPR) domain with three TPR repeats, and a primate unique proline-rich domain at the C-terminus. FKBPs containing TPR domains represent a specific class of immunophilins, a subfamily of chaperones with peptidyl-prolyl cis-trans isomerase (PPIase) activity. This activity interconverts isoforms of proline peptide bonds from *cis* to *trans*, a rate-limiting step in protein folding. These chaperones typically have roles in the transport of protein complexes, translocation, and the formation of receptors and apoptotic complexes^6,7^. However, the active residue required for PPIase activity is absent in AIPL1.

Animal models have provided insights into the role of AIPL1 and revealed normal development but rapid degeneration of rods and cones by four weeks after birth in mice^8,9^. Furthermore, AIPL1 dysfunction associates with the destabilization of a central player in phototransduction, the phosphodiesterase 6 (PDE6) effector enzyme. This holoenzyme, composed of α and β catalytic subunits and two identical inhibitory γ subunits, is the primary regulator of light-dependent changes in cyclic guanosine monophosphate (cGMP). PDE6 is an essential signaling molecule in the visual transduction cascade, and its absence leads to rapid photoreceptor degeneration and vision loss^10–13^. AIPL1 interacts with PDE6α and is essential for the assembly of the PDE6 heteromeric complex in photoreceptors^14,15^. In cones, PDE6 membrane association and assembly is impaired which, together with reduced levels of guanylate cyclase (RetGC), leads to cGMP reduction and cone cell death^9,15^. PDE6α can be modified by a farnesyl lipid group at C-terminal–CAAX box, with this type of prenylation enhancing protein–membrane and protein-protein interactions^16,17^. The mechanism underlying the chaperone activity of AIPL1 involves binding of isoprenyl groups on PDE6α to the FKBP domain of AIPL1. The crystal structure of AIPL1-FKBP demonstrated a uniquely specialized lipid binding motif involving a conformational switch of W72 residue^18^. Additionally, AIPL1 may promote the stability of PDE6 via an interaction with HSP90, thus rescuing PDE6 from proteasomal degradation^19^. AIPL1 also interacts with the NUB1 (NEDD8 Ultimate Buster 1) cell cycle progression control protein by downregulating NEDD8 expression^20^, thus suggesting that the early onset of disease may be a result of the misregulation of photoreceptor development rather than a visual phototransduction defect.

Our current knowledge on AIPL1 derives from animal knockdown and knockout models, and so, we still understand little regarding the molecular mechanism of AIPL1 deficiency in the human context. The unique nature of human retinal development^21^ underscores the need to address the role of AIPL1 in human models to fully decipher the pathogenicity of *AIPL1* mutations and eventually test disease targets and design therapies. The groundbreaking discovery of the Sasai group set the stage for the generation of pluripotent stem cell (PSC)-derived three-dimensional (3D) retinal organoids (ROs) that recapitulate major steps of retinogenesis and self-organize into stratified neural retina with maturing photoreceptor features^22–25^. This approach offers a platform for the exploration of early human retinal development *in vitro* and supports photoreceptor cellular segmentation with nascent light-sensing outer segment (OS) formation within a native retina histoarchitecture that was impossible to achieve in classical two-dimensional cultures or cellular overexpression models. Patient-specific human induced (hi)PSC-derived ROs have also been employed to model retinal diseases, including LCA^26–28^.

In this study, we developed a model of LCA4 by harnessing the potential of patient-specific hiPSCs to recapitulate human retinogenesis in a 3D culture system and interrogated the molecular and cellular events in the absence of functional AIPL1. We used hiPSCs from a patient clinically diagnosed with LCA and molecularly genotyped to harbor a Cys89Arg mutation in AIPL1^29^ that has been proposed to disrupt the farnesyl/FKBP interaction^30^. We induced AIPL1-LCA hiPSCs and control healthy hiPSCs to form ROs to obtain patient-specific primary cells targeted by the disease (i.e., photoreceptors) and explore disease phenotype at the cellular and molecular level. We examined the ability of mutation-bearing ROs to generate the entire retinal cell repertoire in correctly laminated retinal tissue and explored the advanced structural and molecular features of resultant photoreceptors.

## Results

### Efficient generation of AIPL1-LCA ROs from patient hiPSCs

**Figure 1:**
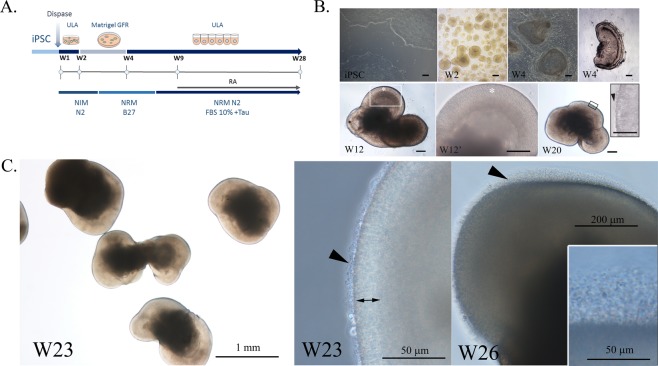
Generation of 3D AIPL1-LCA ROs from Patient hiPSCs. (**A**) Schematic of the differentiation protocol. (**B**) Phase contrast micrographs of differentiation stages: hiPSCs, floating aggregates of hiPSCs following treatment with dispase (W2), and aggregates plated on growth factor reduced (GFR) Matrigel-coated plates reach a typical morphology by week 4 (W4) are dissected manually and are grown in suspension after that (W4’). The typical transparent neuroepithelial domain (*) is formed (W12) with stratified appearance. Inset at larger magnification is shown (W12'). At W20 the projections at the surface begin to emerge (inset, arrowhead). Scale bars: 200 µm. (**C**) By week 23 (W23) the ROs reached 1–1.5 mm in diameter and displayed dense translucent projections at the apical edge (black arrowhead) that grow after that (W26). The double arrow shows the presumptive ONL. Abbreviations: 3D, three-dimensional; RO, retinal organoids; GFR, growth factor reduced; W, week; Tau, taurine; RA, retinoic acid; FBS, fetal bovine serum; ULA, ultra-low attachment plates; ONL, outer nuclear layer.

Mutations in AIPL1 cause autosomal recessive LCA. We employed a previously characterized hiPSC line derived from a LCA patient with a confirmed homozygous mutation in AIPL1 (p.Cys89Arg)^29^ to differentiate as retinal organoids according to a previously published protocol^24^. We employed two hiPSC clones that behaved indistinctly during the differentiation process. Figure 1A depicts a schematic of the retinal differentiation protocol while Fig. 1B,C show phase contrast micrographs taken during various stages of AIPL1-LCA hiPSC differentiation. We also differentiated two control hiPSC lines (Control 1 and Control 2) derived from unaffected individuals under the same experimental conditions and compared results according to the time in differentiating culture, discovering matched morphological changes as demonstrated by light microscopy of Control 1 (Supplementary Fig. S1A). Control 2 exhibited the same morphological changes throughout the differentiation (not shown).

The retinal neuroepithelium formed following manual dissection of optic vesicle (OV)-like structures after 4 weeks (W) of differentiation (Fig. 1B, W4 and W4' and Supplementary Fig. S1A, W4**)** and grew in an apically convex manner after that (Fig. 1B, W12, and Supplementary Fig. S1A, W12). The translucent projections, representing presumptive inner segments (ISs), connecting cilia (CC), and nascent OSs, at the apical edge of the ROs started to appear at W20 of differentiation and became abundant by W23 (Fig. 1C and Supplementary Fig. S1A) similarly to previous reports^31,32^. Protrusions grew over the subsequent weeks, reaching up to 50 µm in length by W26. Excised OVs formed retinal neuroepithelium with an efficiency of 23.6 ± 1.15% (mean ± SD; N = 3 differentiation experiments, n > 200 OVs) for AIPL1-LCA and 23.3 ± 1.52% and 24.3 ± 2.5% (mean ± SD; N = 3 differentiation experiments, n > 200 OVs) in Control 1 and Control 2, respectively (Supplementary Fig. S1B), as judged by light microscopy. These results correlated to histological examinations. The morphology adopted by W26 was maintained upon prolonged culture to W33 (data not shown) in all genotypes.

### Retinal cell specification occurs in AIPL1-LCA and control ROs

We next explored molecular aspects of retinal cell specification in differentiating ROs from the AIPL1-LCA patient hiPSC line and from the two control hiPSC lines (combined as one control because of similar gene expression profiles as described previously^33^), by analyzing transcriptome profiles at four different time points (day (D) 25, D60, D88, and D123 corresponding to W4, W9, W13, and W18, respectively). These time points span retinal organoid formation before the appearance of apical protrusions from ROs. During development, multipotent retinal progenitor cells (RPC) are committed to specific lineage (precursors) and eventually give rise to all six neurons of the retina and Müller glia. The employed differentiation protocol recapitulates retinal neurogenesis, capturing the transient progenitor and precursor states before reaching mature cell features.

**Figure 2:**
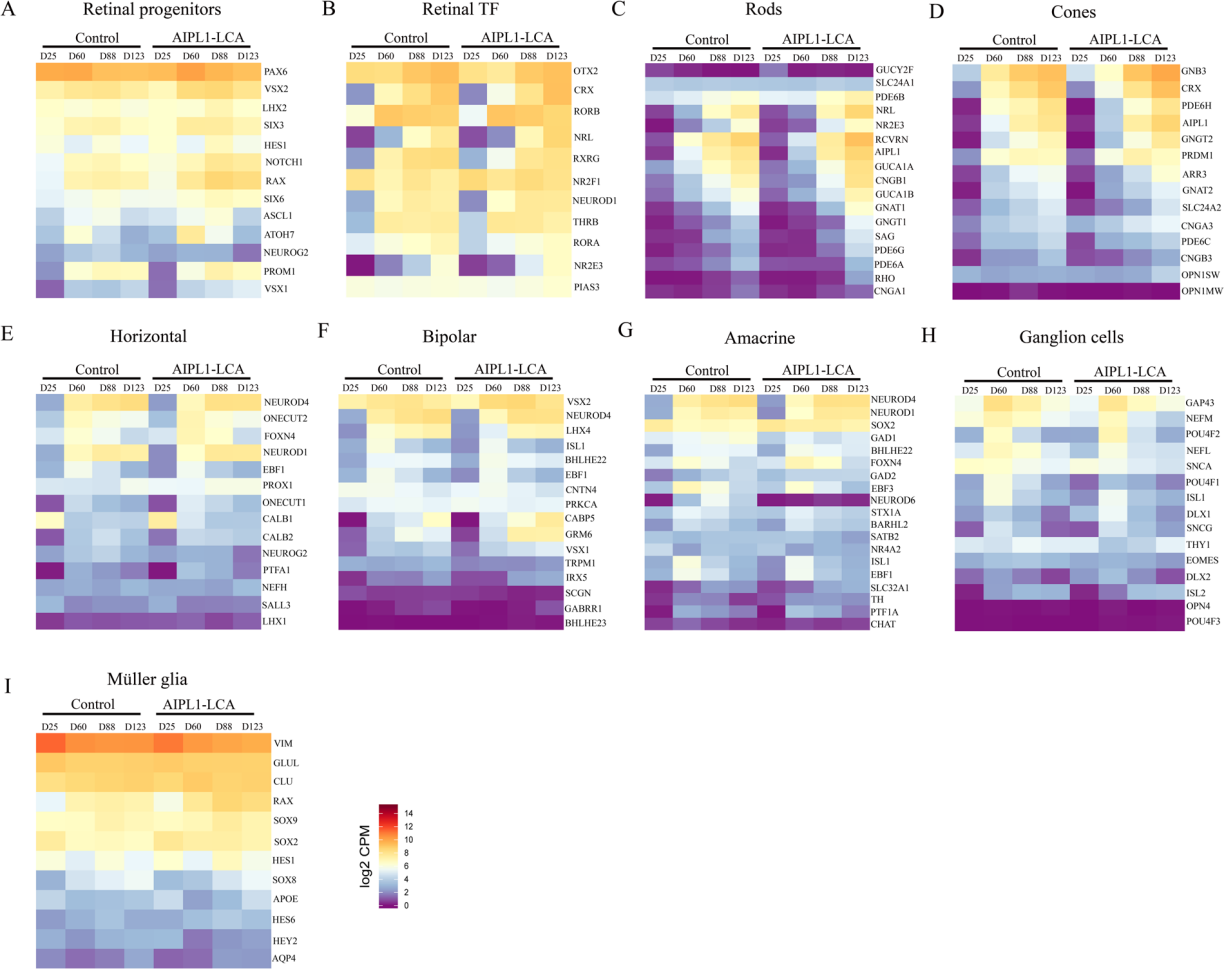
Heatmaps illustrate gene expression profiles of retinal progenitor cells (RPC), retinal transcription factors (TF), and retinal cell type-specific genes at distinct organoid stages from RNA-seq data at day (D) 25, 60, 88, and 123 (corresponding to weeks 4, 9, 13, and 18, respectively) of differentiation. RNA-Seq data show that most genes exhibited comparable expression pattern between control and AIPL1-LCA ROs at respective stages. Different colors represent the expression value as log2 transformed CPM values. Expression of each gene is represented as their average across samples N = 3–5 differentiation experiments with n = 15–20 ROs each. Blue to red represents low to high gene expression.

The raw principal component analyses (PCA) plot (with all expressed genes) indicated that the axis captured the highest variation (17.82%) in the whole transcriptome data corresponds to the differentiation stage of the organoids (Supplementary Fig. S2A). Also control and patient-derived organoids are clearly separated by PC2 (y-axis) (Supplementary Fig. S2A and Fig. S4A). Although PCA showed that AIPL1-LCA samples develop slightly faster, this phase shift was negligible as indicated by retinal cell type markers (Fig. 2). This may suggest that timing of development was similar for both genotypes. In addition, the high RV (Multivariate extension of the Pearson correlation coefficient, called RV coefficient, measures similarity of multiple attributes with multiple features in two datasets^34^) (0.89), the quantitative measure of development time comparison between control and AIPL1-LCA transcriptomes, supported this hypothesis (Supplementary Fig. S2B).

Then, we checked the established cell type –specific genes in the retina (Fig. 2). Figure 2A depicts the expression pattern for RPC markers, which displayed an upregulation in expression of the eye field transcription factors (PAX6, RAX, SIX3, and LHX2), whereas downregulation of expression was evident for RPC genes that are selectively repressed in developing retinas (ATOH7, ASCL1, and NEUROG2). We next explored retina-specific transcription factors and their co-regulators; Fig. 2, panel B shows the early activation of the orthodenticle homeobox 2 (OTX2) photoreceptor precursor cell marker and its downstream target CRX, which is upregulated during photoreceptor development. We observed the expression of NR2E3 and its transcriptional activator NRL (neural leucine zipper) together with their interacting protein PIAS3 according to the correct transcriptional hierarchy of rod differentiation. Furthermore, we noted the later activation of nuclear hormone receptors, such as retinoic acid receptor-related orphan receptor alpha (RORA), another regulator of retinal development and function. The expression of transcription factors required for development, survival, and specification of cone photoreceptors also increased over time (e.g., cone thyroid hormone receptor B (THRB), associated with cone viability, or RXRG, an S-OPSIN suppressor known to be active in immature cones).

We observed the upregulation of other photoreceptor-specific proteins such as AIPL1 and RECOVERIN by W9 (D60) of differentiation and before NRL expression both in control and AIPL1-LCA ROs, in accordance with previous observations in human retinas^21,35^ (Fig. 2C). No change was evident in AIPL1 expression in both developmental schemes, and the single nucleotide missense mutation did not affect alignment quality and quantity (Figs. 2C,D). The expression of mature rod and cone cell markers such as G-protein-coupled receptor RHODOPSIN increased at W18, while we failed to detect cone-specific OPN1MW by W18 (D128) of differentiation (Figs. 2C,D). We observed low levels of OPN1SW expression. We also detected the activation of phototransduction proteins both in rods (PDE6B, PDE6G, PDE6A, GUCA1A, GUCA1B, and CNGB1) and cones (PDE6C, CNGB3, GNAT2, and GNB3) in a similar fashion in control and AIPL1-LCA ROs. The presence of other retinal cell type-specific markers suggested the genesis of horizontal (PROX1), bipolar, amacrine (NEUROD1, NEUROD4) and ganglion cell types (transient expression of POU4F1, POU4F2, ISL1, or increasing expression of THY1) in all ROs (Fig. 2E–I).

The only exception to the overall similarity in expression profiles between the control and AIPL1-LCA ROs was in *NEUROD6* (Fig. 2G), a helix-loop-helix transcription factor involved in GABAergic amacrine cell subtype specification^36^. We failed to observe *NEUROD6* expression in AIPL1-LCA ROs.

Taken together, these data suggest the activation of the molecular networks required for retinal cell specification, which follows the chronological cell birth sequence of the native retina for both healthy and AIPL1-LCA ROs.

Differential expression (DE) analyses between the time points, within each group separately, revealed at least 2-fold levels of alterations in 3794 genes, with 2182 genes common between groups (Supplementary Fig. S3A). 797 genes and 815 genes showed response to time either in control or patient derived ROs respectively (Supplementary Fig. S3A). The gene ontology (GO) terms of intersecting genes (Supplementary Fig. S3B) reflect a summary of GO terms belonging to clusters having a similar developmental pattern in both datasets (Supplementary Fig. S3C,D). GO identified enrichment in genes associated with mitotic cell division, signal release from the synapse, and visual perception suggesting that the main steps of commitment toward retinal cell fates occur in both genotypes. The number of DE genes with respect to time unique to each group indicated differences between both development schemes even though the core development is very comparable. The genes unique to the control group (797) are enriched in neuronal connection-formation related GO terms (Supplementary Fig. S3B). AIPL1-LCA mutant-specific genes belonged to two GO terms; one related to antigen possession for immune responses, and the other related to microtubule functioning (Supplementary Fig. S3B). The transcriptomic differences between the two groups could be used to direct appropriate downstream experiments.

Moreover, clustering of all the time-responsive DE genes revealed 12 clusters, (C1-C12), with some differences between the two groups (Supplementary Fig. S3C). Four clusters showed gene expression changes that are likely caused by AIPL1 mutation in patient organoids. C1 genes are turned off earlier in the control, whereas their down regulation during maturation is slower in the patient-derived retinal organoid samples. Genes in C7 exhibit increasing expression with time in the patient organoids but not appreciably in controls. Expression of C8 genes peaks at D88 in the control group; however, their expression continues to be high at D123 in the patient group. C9 genes are showing increasing expression with developmental time in control group, but not in the patient organoids.

Although the differences in gene expression profiles had no apparent effect on normal differentiation of retina, especially on the formation of the OS, DE genes unique to either group showed substantial disruption on time matching compared to common DE genes indicated by RV coefficients 0.69 and 0.95, respectively (Supplementary Fig. S4B,C). Furthermore, the captured meaningful variation (related to the developmental time) by PCA of common DE genes was not much higher than that of all expressed genes (less than 9%, 26.69%, and 17.82%, respectively) (Supplementary Fig. S2A and S4A).

We also studied the differentiation progress of AIPL1-LCA hiPSCs by immunofluorescence. We examined the genesis of retinal cell types by following the expression of a panel of major retinal cell type markers spanning the 27 weeks of differentiation (Supplementary Fig. S5). hiPSC-derived ROs followed the temporal sequence of expression of human retinal cells^24^, namely ganglion cells followed by photoreceptor progenitors, amacrine, and horizontal cell types. Towards the end of the differentiation process, photoreceptors mature and are then followed by the appearance of bipolar cells  and Müller glia. We detected ganglion cells by NEUN staining at W9, followed by the OTX2 and CRX early photoreceptor progenitor markers, which were restricted toward the presumptive outer nuclear layer (ONL) on the apical border at later stages. We detected the expression of NRL, the earliest rod-specific marker, at W13 in 33,3 ± 5% of the ROs (16 ROs analyzed, n = 3 differentiation experiments). The AP2 pan amacrine cell marker began to be expressed before W10, while CALRETININ positive immunostaining, specific for the type II subtype, appeared later at W13. We observed the CALBINDIN horizontal cell marker basally at W13 followed by an increase in the number of cells and the segregation to the presumptive intermediate layer. We also observed fluorescence signal at the apical surface of the ROs, which we presume to be cones, as CALBINDIN can also label cone cells^37,38^. Müller glia exhibited a weak cellular retinaldehyde-binding protein (CRALBP) staining by W17 but high expression at W21, with the characteristic extended feet across the organoid. The bipolar cells were born last and were not detected until W25 (not shown).

Overall, the time course of retinal cell genesis in the absence of functional AIPL1 followed the conserved sequence of emergence of vertebrate retinal cells, with ganglion cells being born first, followed by photoreceptor precursors, horizontal, amacrine, and later by Müller glia, bipolar cells, and maturing photoreceptors. We observed the organization of retinal cell types along the apico-basal axis in agreement with their distribution in the native retina.

### AIPL1-LCA ROs exhibit correctly-organized ONL and support synaptic contact formation

**Figure 3:**
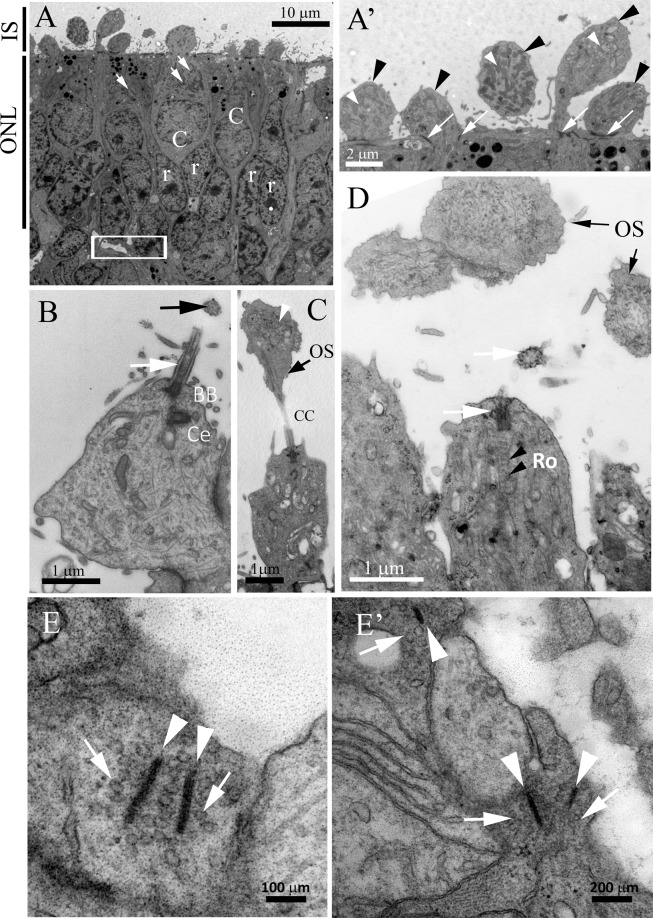
Representative Electron Micrographs of AIPL1-LCA ROs. (**A**) Ultrastructure of the ONL-like region: ISs protruding at the apical side of the organoid, cone-like cells (C) at the outermost side of the ONL with large cell body and large bright nucleus and cytoplasmic mitochondria (white arrow). Deeper aligned, presumably rod photoreceptors with electron dark nuclei (r). White square showing the area where synaptic ribbons identified. (**A’**) Higher magnification of ISs (black arrowheads) at the apical border with elongated mitochondria (white arrowheads) and OLM (white arrows). (**B**) Longitudinal section of CC (white arrow) arising from the ISs with the BB at the base and the Ce at a right angle below the BB. The transversal section of the CC reflecting microtubules arranged to form a cylinder (black arrow). (**C**) CC protrudes into organelle-free vesicle with membranous structure precursors of the disc stacks (white arrowhead) of the OSs. (**D**) CC arising from the IS and a transversal section of the CC lying in the area outward of the IS (white arrows). Dark cross-striation representing rootlet filaments (black arrowheads, Ro) protruding from the BB toward the cytoplasm. Organelle free vesicles, presumably presenting nascent OSs (black arrows), observed in the vicinity of the CC outward of ISs. (**E**,**E’**). Multiple synaptic ribbons with electron dense bars (white arrowheads) surrounded by presynaptic vesicles (white arrows). Abbreviations: ONL, outer nuclear layer; ISs, inner segments; OLM, outer limiting membrane; CC, connecting cilium; BB, basal body; Ce, centriole; Ro, rootlets; OS, outer segment.

We next explored the ultrastructure of ROs derived from AIPL1-LCA hiPSCs and compared them to control ROs using transmission electron microscope (Fig. 3 and Supplementary Fig. S6**)**. Analysis of the apical surface of ROs at W27 revealed an ONL-like layer with distinguishable cone-like cells with large cell bodies and clear large nuclei lying adjacent to the apical border (Fig. 3A). We detected dark nuclei pertaining to presumptive rods in several stratified layers deeper in the ONL-like layer (Fig. 3A). We also observed protruding mitochondria-rich ISs, reminiscent of the mature ellipsoid in the native retina, at the apical edge (Fig. 3A’). The ISs presented CC and basal body (BB) with the typical microtubule organization parallel to the CC axes and juxtaposed centriole (Ce) (Fig. 3B). Occasionally, the CC protrude into vesicles with membranous structures, the precursors of the disc stacks reminiscent of organelle-free OSs (Fig. 3C). Typically, the rudimentary OSs could not be traced to their CC and ISs probably due to the small CC diameter which is not captured in fine microscopic sections (Fig. 3D). The examination of the retinal organoid sections revealed photoreceptor synaptic ribbons, the structural specialization of ribbon synapses, in the area below the dark presumably rod nuclei (Fig. 3A, inset**)**. The synaptic ribbon could be distinguished as an electron dark bar with docked presynaptic vesicles lying orthogonally to the presynaptic membrane (Fig. 3E-E’). The presence of a single bar is characteristic of rod spherules, while cone pedicles exhibited multiple circularly arranged bars tethering toward the same presynaptic membrane (not shown).

Overall, the ultrastructural examination revealed similar mature features found in AIPL1-LCA and control ROs of the same differentiation stage (Supplementary Fig. S6), also showing similarities to previous reports^31,32^.

### Photoreceptors in AIPL1-LCA ROs express mature photoreceptor markers

**Figure 4:**
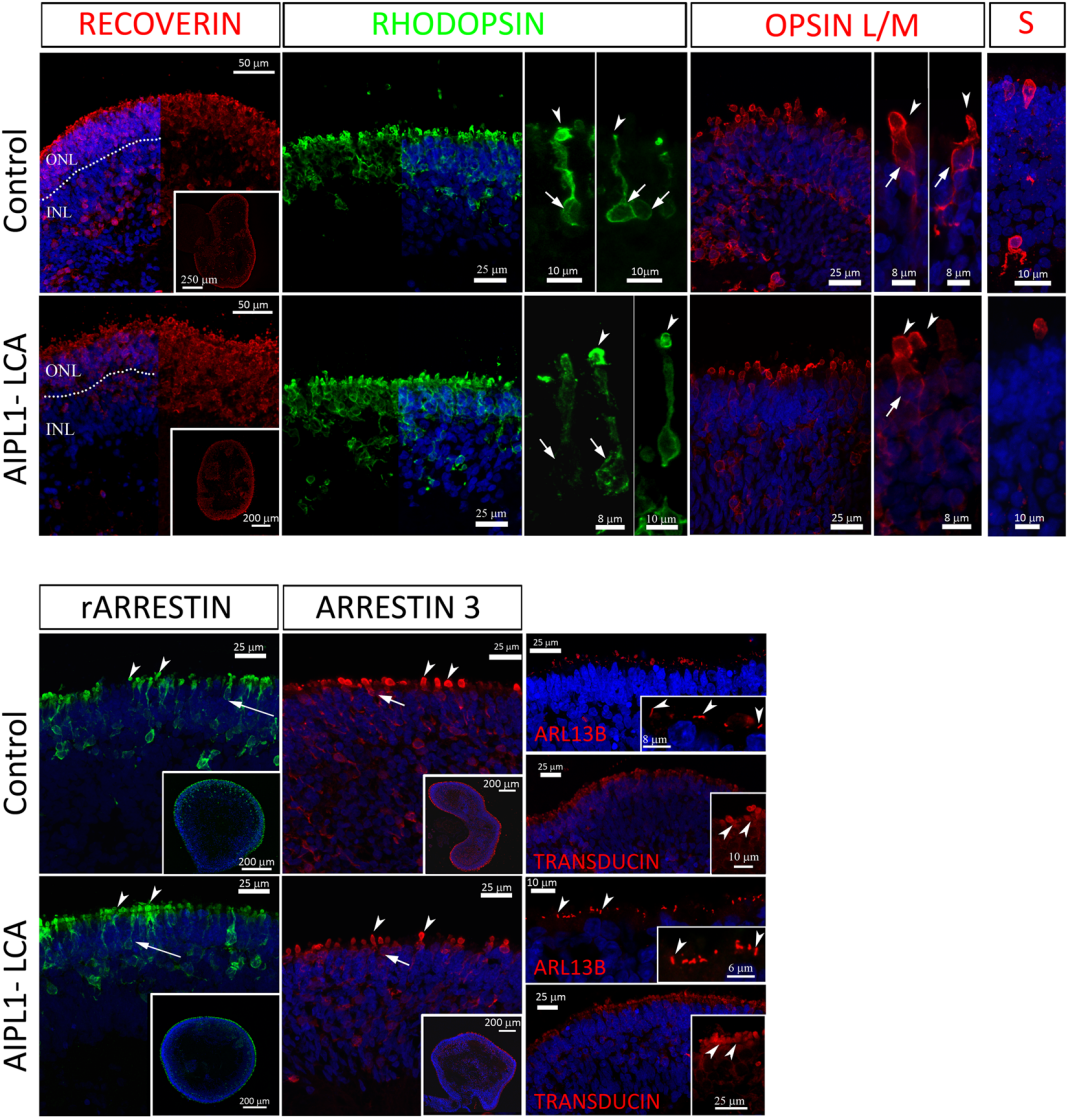
Immunofluorescence staining for mature photoreceptor cell markers in cryosections of AIPL1-LCA and Control ROs after 27 weeks of differentiation. Immunohistochemistry of RECOVERIN (dotted line indicates the gap between the ONL and INL, inset showing low magnification image), OPSIN L/M, OPSIN S (S), Rod ARRESTIN (rARRESTIN, inset shows lower magnification image), ARRESTIN 3 (inset shows lower magnification image). Arrowheads indicate ISs and arrows indicate photoreceptor cell bodies. ARL13B is a connecting cilia marker. Inset showing higher magnification of labeled cilia (arrowheads). α-TRANSDUCIN labels rod photoreceptor cells. Inset showing higher magnification of stained cells. Arrowheads indicate OLM narrowing beyond the ISs. Nuclei were counterstained with DAPI (blue). RECOVERIN and RHODOPSIN panels show DAPI staining at the left and right half, respectively. All data labelled as Control are from Control 1 ROs; Control 2 behaved indistinctly. Abbreviations: RO, retinal organoids; ISs, inner segments; OLM, outer limiting membrane, INL, inner nuclear layer; ONL, outer nuclear layer.

To test whether the mutation in AIPL1 affects the expression of major mature photoreceptor markers, we examined a large number of photoreceptor-specific proteins for their levels of expression and subcellular distributions by immunofluorescence in AIPL1-LCA ROs. Figure 4 establishes the apical expression of all photoreceptor-specific proteins in the presumptive ONL layer at W27 of differentiation. We detected RECOVERIN apically, staining the entire retinal organoid apical border, and in isolated cells at the outer edge of the inner nuclear layer (INL), presumably staining bipolar cells. We first detected RHODOPSIN, a selective marker for rods, at W21 of differentiation in restricted regions in AIPL1-LCA and control ROs (not shown). During later stages of differentiation, RHODOPSIN showed positive staining along the apical edge with characteristic membrane staining revealing a typical elongated rod cell body (arrow) localized deep in the ONL-like layer, and accumulation in ISs (arrowhead). L/M OPSIN positively stained cone cells with larger stubby cell bodies (arrow) adjacent to the presumptive outer limiting membrane (OLM) and ISs protruding outward (arrowhead). We rarely observed S OPSIN in patient and control samples, and in both cases, any staining was localized to individual cells at the apical edge of the organoid. We occasionally observed positively stained cells deeper in the organoid, reflecting cells that have not yet reached their final allocation (arrowhead). We detected rod ARRESTIN expression in rod-like cells aligned to the presumptive ONL-like layer. Rod ARRESTIN staining revealed a typically segmented rod with ISs (arrowhead) protruding beyond a presumptive OLM and deeply localized elongated cell body (arrow). ARRESTIN 3, a pan cone marker, stained larger ISs (arrowhead) and cone cell bodies (arrow) at the apical edge of both AIPL1-LCA and control retinal organoid sections and cells localized below the presumptive ONL, presumably cones that have not yet reached the apical location.

To investigate the development of cilia, we stained for the ARL13B ciliary marker in both ROs, observing staining at the apical surface. The rod-specific TRANSDUCIN-α subunit and RetGC (not shown) phototransduction proteins were also expressed in the presumptive ONL-like layer positively staining the ISs and less intensely the cell bodies (inset).

Thus, we did not detect any significant differences with regards to the expression of photoreceptor-specific markers and their localization between AIPL1-LCA and control ROs.

### Lower expression levels of AIPL1 and PDE6α in AIPL1-LCA ROs

**Figure 5:**
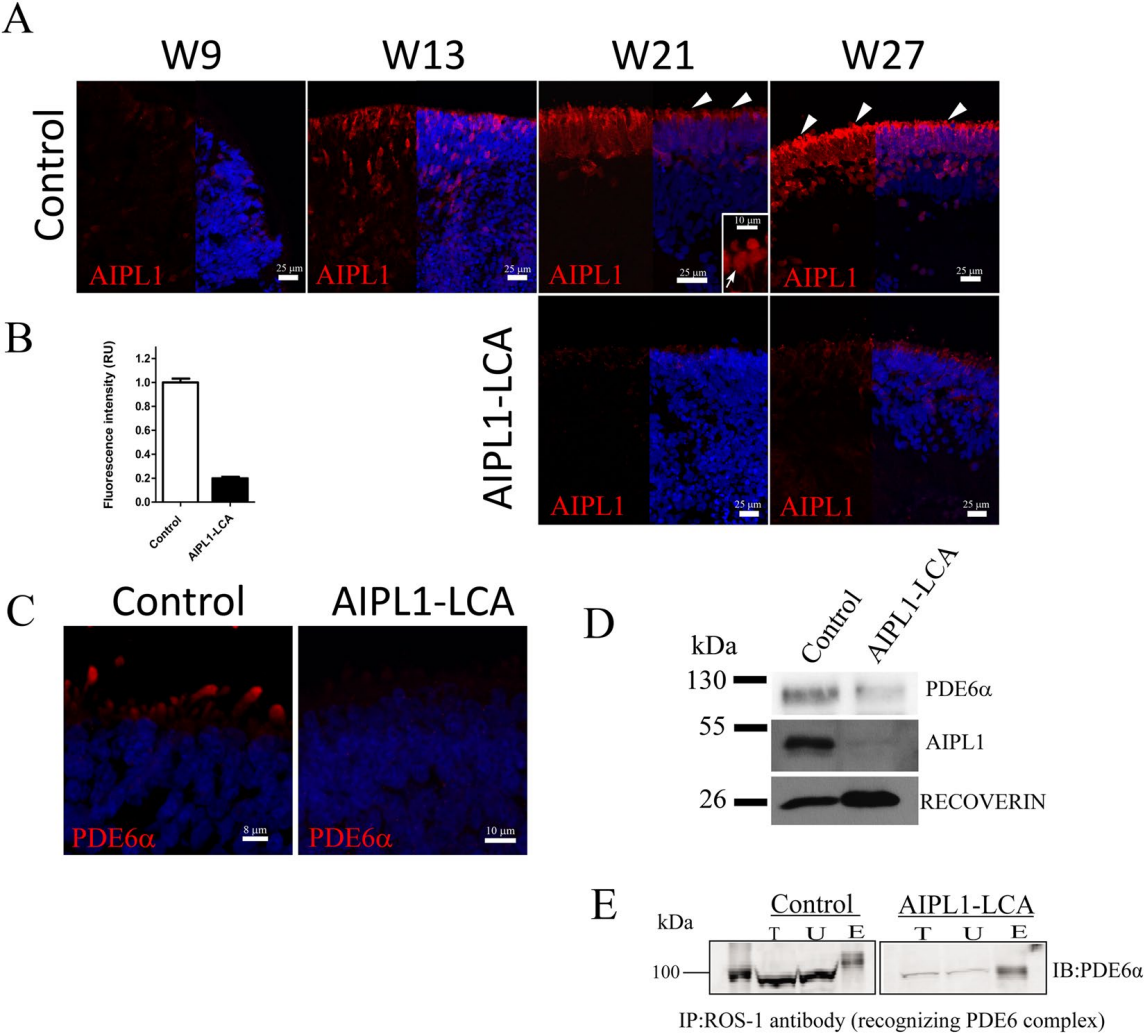
AIPL1 and PDE6α Expression in Control and AIPL1-LCA ROs. (**A**) Immunohistochemistry of AIPL1 at W9, W13, W21, and W27 in Control and AIPL1-LCA ROs. AIPL1 can be detected as early as W9 of differentiation in control ROs; AIPL1 shows scattered staining across the NR at W13. At W21 and W27 AIPL1 + cells align apically in the ONL-like region. Weak staining in the ONL-like region can be appreciated at week 21 and 27 in AIPL1-LCA ROs. The right half of the images show nuclei counterstained with DAPI (blue). (**B**) AIPL1 relative fluorescence intensity in control and AIPL1-LCA ROs at W27 cryosections (mean ± SD, n = 16 ROs from N = 3 differentiation experiments each, Unpaired student *t* test, ***P < 0.001). (**C**) Immunohistochemistry of PDE6α in Control and AIPL1-LCA ROs at W27 of differentiation. Nuclei were counterstained with DAPI (blue). (**D**) Western blotting of AIPL1 (MW 43 kDa), RECOVERIN (MW 26 kDa) and PDE6α (MW 99 kDa) expression in W33-old patient and Control 1 ROs. (**E**) Immunoprecipitation with ROS-1 antibody (IP) and immunoblotting with PDE6α (IB). PDE6α expression shown in T (total protein in the ROs lysate), U (Unbound fraction), E (Elution). Full blot images shown in Supplementary Fig. S7B and S7C. All data labelled as Control are from Control 1 ROs. Abbreviations: RO, retinal organoid; ONL, outer nuclear layer; NR, neural retina; W, week.

We finally employed immunofluorescence to examine AIPL1 expression at different stages during the differentiation protocol for control and AIPL1-LCA ROs. Figure 5A demonstrates the early expression of AIPL1 at W9 in the control sample, as faint staining at the apical-most part of the organoid. At W13, the ROs exhibited disperse staining across the neuroepithelium. AIPL1 positive cells accumulated at the apical side by W21, showing cytoplasmatic staining of ISs (arrowheads), soma (arrow), and axons as described in native retina^39^. We observed more prominent staining by W27 at which point we observed that the entire organoid was lined by AIPL1 positive staining at the apical border.

In contrast, AIPL1-LCA ROs displayed only weak staining at W21 and W27 under the same experimental conditions. The AIPL1 signal was significantly reduced between patient and Control 1 ROs at W27 (Fig. 5B). We verified the expression of AIPL1 by immunoblotting, and in line with immunofluorescence data, we found a dramatic reduction in expression of AIPL1 in patient-derived ROs as compared with control ROs collected at W27 of differentiation (Supplementary Fig. S7A**)** and in W33-old ROs (Fig. 5D and Supplementary Fig. S7B).

Animal models had shown that AIPL1 deficiency causes destabilization of PDE6 by preventing its assembly, which leads to rapid protein degradation^8^. In the native retina, PDE6 complex is synthesized and assembled in the cytosol of photoreceptor IS, and subsequently traffics to the OS, the site of phototransduction cascade^40^. We performed immunofluorescence to detect PDE6α in ROs and detected positive staining in a portion of control ROs (26,6 ± 5,7% N = 17 ROs, n = 3 differentiation experiments) in the ISs. None of the AIPL1-LCA ROs displayed PDE6α signal at the apical surface at the same differentiation stage (Fig. 5C). We corroborated the reduced levels of PDE6α by immunoblotting W27 and W33 ROs (Supplementary Fig. S7A and Fig. 5D). We also assayed for PDE6 heteromer assembly using a previously characterized monoclonal antibody, ROS-1, that recognizes a fully assembled PDE6 complex. Our pull-down with ROS-1 in both AIPL1-LCA and control ROs revealed the presence of assembled PDE6 **(**Fig. 5E and Supplementary Fig. S7C**)**.

Thus, in line with the AIPL1 animal model data^8,14,41^, we observed reduced levels of the AIPL1 primary target protein PDE6α, in AIPL1-LCA RO photoreceptors. Of note, the reduction in AIPL1 and PDE6α protein levels were not caused by a reduction in their mRNA levels (Supplementary Fig. S8).

Therefore, AIPL1-LCA ROs exhibited a reduction in the levels of AIPL1 protein and decreased PDE6α expression, whereas other examined photoreceptor markers remained unaltered. Altogether, our findings show that despite reduced levels of AIPL1 and PDE6, the assembly of PDE6 heteromer is not affected in AIPL1-LCA ROs.

## Discussion

In this study, we set out to identify the early events in the development of LCA4 and understand disease pathology in the patient’s genetic background. Previous studies described AIPL1 expression early in development, at fetal week 11.8 in central human retina^39^, at the fetal age of 59 days, concurrent with the expression of OTX2^21^ and NRL, a master regulator of rod photoreceptor differentiation^35^. Given the early expression of AIPL1 during development and the early clinical onset of AIPL1-caused vision loss, we rationalized the recapitulation of retinogenesis in a retinal organoid model as the most appropriate means to study disease-related events.

We provide evidence that AIPL1-LCA ROs exhibit a similar morphology to that of healthy control ROs during differentiation. Specifically, AIPL1-LCA ROs exhibit a similar efficiency in neuroepithelium formation with the concomitant appearance of apical protrusions during W20 of culture that reaches up to 50 µm in length in both genotypes by W26. The concurrent differentiation dynamics were also evidenced by gene expression analysis that supported temporally-matched profiles for AIPL1-LCA and control ROs. Transcriptional dynamics during retinal differentiation were similar between the two genotypes, as indicated by the RV coefficient of 0.89 and this generally agreed with what we currently understand about mammalian retinal development, such as the hierarchical activation of photoreceptor precursor markers (OTX, CRX, RECOVERIN) or rod and cone-specific markers (NRL, NR2E3, GNGT1, PDE6H, GNGT2, GNAT2)^21,42,43^. AIPL1-LCA ROs formed main retinal cell types (photoreceptors, bipolar, ganglion, amacrine, horizontal cells, and Müller glia) ordered in a correctly stratified native retina-like manner with photoreceptors aligning to the presumptive ONL, apical-most distributed cones, and deeper allocated rods in several layers. Horizontal and amacrine cells migrated toward their corresponding location in the presumptive INL, while the ganglion cell layer was confined to the basal edge. Müller glia spanned the entire neuroepithelia with characteristic feet extending beyond the photoreceptor somata to the OLM. We observed synaptic contacts in the presumptive outer plexus underneath the dark rod nuclei with typical retinal ribbon synapse ultrastructure. Moreover, the ultrastructural features of photoreceptors, such as the formation of ISs, cilia and developing OSs, were similar to the control ROs. Pan photoreceptor and cone- and rod-specific marker immunoreactivity did not show significant differences in expression pattern.

We observed differences between AIPL1-LCA and control ROs with regards to immunoreactivity to AIPL1, with lower staining in the patient hiPSC-derived ROs compared to control ROs. The reduction in levels of AIPL1 p.Cys89Arg is likely due to misfolding of AIPL1 and consequent degradation. This finding contrasts with the exogenously overexpressed AIPL1 p.Cys89Arg and other missense mutants whose expression was similar to wild-type AIPL1 in HEK293T and COS-7 cells^9,19^. We hypothesize that this difference in mutant AIPL1 stability can be attributed to the heterologous expression system under the exogenous, strong promoter that evades endoplasmic reticulum surveillance or that photoreceptors employ more stringent quality control systems. This finding highlights the importance of studying protein fate in the physiologically relevant cellular environment.

In keeping with the known role for AIPL1 in regulating the levels and assembly of PDE6, our study demonstrated a reduction in PDE6α levels in AIPL1-LCA ROs. mRNA expression levels for AIPL1 and PDE6α remained unaltered in AIPL1 Cys89Arg genotype, corroborating the findings in mouse models and suggesting that AIPL1 acts post-translationally on PDE6α.

Despite the associated clinical phenotype being characterized by early-onset severe visual loss in patients bearing the *AIPL1* c.256 T > C mutation, we were unable to detect overt photoreceptor degeneration in this model. We also failed to observe reactive gliosis, as assessed by GFAP expression, and increased cell death, as assessed by apoptotic markers, in AIPL1-LCA ROs (data not shown). The ultrastructural examinations failed to detect early signs of photoreceptor degeneration, such as shorter ISs, OSs, nuclear disorganization or IS vacuolar inclusions as observed in animal models^8^. These observations can be attributed to several concurring events.

First, the ROs support photoreceptor maturation up to the formation of nascent OSs. The ultrastructural examination showed OS-like vesicles with membranous formations inside, reminiscent of stacked membrane disks both in AIPL1-LCA and control RO photoreceptors after 27 weeks in culture. We did not observe further maturation after that, including OS elongation or membranous discs stack formation. PDE6α, the primary AIPL1 interacting protein, localized to photoreceptor ISs probably due to incomplete formation of OSs, where it resides in mature photoreceptors. Such rudimentary OSs formation has been observed in native rods in fovea at fetal week 26^44^, matching the temporal stage in this *in vitro* model. Therefore, the phototransduction machinery has not been activated yet with consequently unaltered cGMP levels, the main trigger of photoreceptor cell death. Despite many efforts, the incomplete formation of OSs in this retinal organoid model has yet to be overcome, either by prolonged cell culture or through direct contact with an RPE layer.

Second, high-resolution optical coherence tomography in the foveal region in young LCA4 patients suggests that the outer retina may be preserved in patients up to one year of age^45^. These observations are in concordance with the proposed “biochemical dysplasia” category of LCA, described by Koenekoop *et al*., since patients‘ retinal outer and inner layer appear intact in the presence of defective AIPL1, in contrast to “aplasia” and “degeneration” subtypes of the disease^46^.

This first human LCA4 model identifies the reduction of AIPL1 levels as the earliest disease event while ROs preserve inner and outer retinal morphology at least until the stage of OS formation. This finding excludes the hypothesis that AIPL1 is necessary for rod and cone development and supports the notion that AIPL1 function is linked to phototransduction activity in photoreceptors. The generation of patient-specific diseased tissue via hiPSCs allows for the generation of human disease models without genetic manipulation and represents an unprecedented resource to study clinically relevant phenotypes and cell therapy approaches. So far, hiPSC LCA models have addressed the deficiency in ciliary protein CEP290 required for ciliogenesis and ciliary traffic control, the structural component that could be followed by fluorescent labeling or ultrastructural exploration. The present study addresses the modeling of phototransduction protein defects whose phenotype may be fully captured by further maturation of photoreceptors and functional analyses including light exposure to initiate the visual cascade.

This model underscores the features captured by model animals, i.e., lower levels of AIPL1 and PDEα, as well as aspects that require additional consideration to phenocopy the disease fully. The particularly suitable short size of the *AIPL1* gene supports gene therapy approaches^47–49^, and this personalized model could present an excellent resource in which to test different optimization strategies. Fine tuning of adeno-associated virus transfer vector design or dosing tests will be necessary to improve therapeutic gene expression in human photoreceptors, and this approach represents a unique clinically relevant resource.

## Materials and Methods

### hiPSC lines, cell culture and retinal differentiation

The cell lines employed in this study include the hiPSC cell line (LCA-FiPS4F1)^29^ from the LCA patient homozygous for the mutation in AIPL1, p.Cys89Arg (c.265 T > C) and two control hiPSC cell lines: Ctrl2-FiPS5F2 (Control 1)^50^ and Ctrl1-FiPS4F1 (Control 2), were subjected to the same differentiation conditions in parallel. All cell lines are deposited in the Spanish National Stem Cell Bank (www.isciii.es/QueHacemos/Servicios/BIOBANCOS/BNLC/).

hiPSCs were cultured in plates coated with BD Matrigel^TM^, human embryonic stem cell-qualified Basement Membrane Matrix (Corning) using mTeSR1 medium. Passages were performed using Dispase (STEMCELL Technologies, #07913), every 5–7 days at 1:6–1:10 split ratio. Quality controls such as mycoplasma contamination, karyotype verification, and cell line authentication were regularly performed.

The hiPSC lines were induced to differentiate toward 3D neural retina following the protocol published by Zhong *et al*.^24^. The hiPSC were grown to 60–80% confluency and then lifted with dispase or EDTA, pipetted up and down and transferred to ultra low attachment (ULA) plates for 1 week in neural-induction medium (NIM) (DMEM/F12 (1:1), 1x N2 supplement (Invitrogen), 1X MEM non-essential amino acids (NEAA), 2 µg/ml heparin (Sigma)). The floating aggregates were then transferred to plates coated with BD, Matrigel^TM^-Growth Factor Reduced (GFR) (Corning) and left to grow for 2 weeks. The OV-like structures were excised with the needle and cultured individually in ULA 96 well plates in neural retina medium (NRM, DMEM:DMEM/F12 (2:1), 1x B27 (Invitrogen), 1X NEAA, and 1% antibiotic–antimycotic). At day 42 of culture, the NRM was supplemented with 10% FBS (Gibco), Taurine (100 µM), all-trans retinoic acid (1 µM). From day 93 the retinoic acid was reduced to 0,5 µM.

### Immunohistochemistry

hiPSC-ROs were fixed in PFA 4% for 30 min at room temperature (RT). Thereafter the ROs were introduced into 10%, 20% and 30% sucrose successively O/N at 4 °C and included into OCT. Histological sections, 10 µm thick, were performed using Microm HM 505E cryostat. Antibodies used are described in Supplementary table S1. DAPI (40,6-diamidino-2-phenylindole) was used for nuclear counterstaining. Slides were mounted with Vectashield (Vector Laboratories). The images were taken on Leica SP8 confocal microscope with HC PL APO CS2 63×/1.40 OIL and HC PL APO CS2 40×/1.30 OIL objectives and processed by Leica LAS AF, Photoshop CS2 (Adobe), and Image J software. All presented images labelled as Control are from Control 1. No differences in retinal cell type expression and distribution were observed between Control 1 and Control 2.

### Transmission electron microscopy

hiPSC-ROs were fixed in 4% paraformaldehyde, 2,5% glutaraldehyde in 0.1 M sodium phosphate buffer (pH 7,2–7,4) for 1,5 h and washed with the same buffer. Then, the samples were post-fixed with 2% osmium, rinsed, dehydrated and embedded in Durcupan resin (Fluka, Sigma-Aldrich, St. Louis, USA). Serial semi-thin sections (1.5 µm) were cut with an ultramicrotome Ultracut UC-6 (Leica, Heidelberg, Germany), mounted onto slides and stained with 1% toluidine blue in 1% Borax and analyzed under a light microscope. Ultrathin sections (0.07–0.09 µm) were prepared with the Ultracut and stained with lead citrate. Finally, photomicrographs were obtained under a transmission electron microscope FEI Tecnai G2 Spirit (FEI Europe, Eindhoven, Netherlands) using a digital camera Morada (Olympus Soft Image Solutions GmbH, Münster, Germany). Sample processing was performed by CIPF Electron Microscopy Core Facility.

### Western blot and Immunoprecipitation (IP)

Control 1, Control 2 and AIPL1-LCA ROs collected at W27 (N = 2) and Control 1 and AIPL1-LCA ROs collected at W33 (N = 2) were lysed in RIPA buffer (R0278 Sigma) containing a protease inhibitor coctail (Roche), and total protein was quantified using a Bradford Reagent protein assay (B6916 Sigma-Aldrich). Protein lysates were denatured by 1X SDS Sample Buffer. The resulting samples were incubated at 95 °C for 5 min. Protein samples (20–30 µg) were then separated on TGX Stain-FreeTM Gels (BioRad), and electroblotted onto a PVDF membrane (Trans-Blot® TurboTM Transfer Pack/ Bio-Rad). Membranes were cut horizontally (at ladder bands corresponding to MW 72 kDa, and 34 kDa) into 3 strips, incubated in blocking buffer (5% nonfat dried milk diluted in TBS + 0,1% Tween) for 1 h at room temperature, washed three times in TBS + 0.1% Tween for 5 min and each strip incubated with corresponding primary antibody (AIPL1, 1:10.000, RECOVERIN 1:1000 and PDE6α 1:1000) in blocking buffer overnight at 4 °C. Thereafter, blots were washed three times in TBS + 0.1% Tween and incubated with secondary HRP-conjugated antibody in blocking buffer for 45 min at RT. Blots were washed another five times and protein bands were visualized using SuperSignal West Pico PLUS (Thermo Scientific) on X-ray films (AIPL1, RECOVERIN, PDE6α) and G.BOX Chemi XX6 (SYNGENE) system (PDE6α).

To analyze effect of AIPL1 C89R mutation on PDE6 complex assembly, immunoprecipitation (IP) was performed with antibody against ROS-1. This was followed by immunoblotting (IB) with PDE6α antibody. 25 Weeks retinal organoids derived from controls and AIPL1-LCA patient were lysed in 200 µl of lysis buffer (50 mM Tris.Cl-pH 7.4, 300 Mm NaCl, 5 Mm EDTA and 0.02% Sodium Azide) containing Protease inhibitor pellet, 10 mM of iodoacetamide and 1% triton X-100. After centrifugation at the speed of 14000 × g for 30 sec at 4 °C; the supernatant (T) was incubated with 5 µg of antibody (ROS-1) for 2 h at 4 °C followed by incubation with 10 µl of A/G agarose beads for 3 h at 4 °C. The Unbound proteins (U) were removed by centrifugation at 14000 × g for 30 sec at 4 °C. The beads were washed three times with 1 ml of the wash buffer (0.1% triton x-100, 50 mM Tris.Cl-pH 7.4, 300 Mm NaCl, 5 Mm EDTA and 0.02% Sodium Azide) and a final wash in 1 ml of 1 × PBS. After the wash, the samples were eluted to a final concentration of 1X SDS sample buffer (4x Tris-Cl/SDS pH6.8, 30% glycerol, 10% SDS, 0.6 M DTT and 0.012% bromophenol and β-mercaptoethanol). The samples were boiled for 5 min. 10 µL of each sample were loaded on SDS PAGE gel and then immunoblotted using PDE6α antibody diluted in 1:1 ratio of blocking buffer (Licor) and 1X PBST (1:1000). The blot was imaged using Li-cor imaging system. This experiment was repeated three times with different retinal organoids samples each time.

### Ethical approval

The study was approved by the corresponding Ethics Committee CAEC (Comité Etico Autonomico de Estudios Clínicos), Valencia, Spain. Informed consent was signed by the involved human subjects. All procedures were done in accordance with institutional guidelines and regulations.

## Supplementary information

Supplementary Information.

## Data Availability

All raw and processed data are available through Gene Expression Omnibus GSE131877 (www.ncbi.nlm.nih.gov/GEO).
