# Supplementary material for: Retinal Organoids derived from hiPSCs of an AIPL1-LCA Patient Maintain Cytoarchitecture despite Reduced levels of Mutant AIPL1
Source: Sci Rep. 2020 Mar 25;10:5426. doi: 10.1038/s41598-020-62047-2 (PMC7096529; doi:10.1038/s41598-020-62047-2)
Supplement: Supplementary file 1 — Supplementary Information. [file 41598_2020_62047_MOESM1_ESM.pdf]

Retinal Organoids derived from hiPSCs of an AIPL1-LCA Patient Maintain Cytoarchitecture despite Reduced levels of Mutant AIPL1

Dunja Lukovic[1,2]*, Ana Artero Castro[2], Koray Dogan Kaya[3], Daniella Munezero[4], Linn Gieser[3], Carlota Davó Martínez[2], Marta Cortón[5], Nicolás Cuenca[6], Anand Swaroop[3], Visvanathan Ramamurthy[4], Carmen Ayuso[5] and Slaven Erceg[2,7]

SUPPLEMENTAL INFORMATION

Supplemental experimental procedures

Supplementary figures

Figure S1. Differentiation of control hiPSC lines toward ROs

Figure S2. Projection of the raw data into two dimensions.

Figure S3. DE analysis with respect to time in both transcriptomic data

Figure S4. Projection of the data into two dimensions with DE genes.

Figure S5. Time course of retinal cell marker expression during the differentiation of AIPL1-LCA organoids

Figure S6. Representative electron micrographs of control ROs at W27.

Figure S7. Supporting western blot images for Figure 5D and 5E

Figure S8. RT-qPCR analysis of *AIPL1* and *PDE6α* expression in retinal organoids.

Supplementary tables

Table S1. List of used antibodies

Table S2. List of TaqMan® Gene Expression ID Assays used for qRT-PCR

SUPPLEMENTAL EXPERIMENTAL PROCEDURES

LCA Patient Clinical Phenotype

The patient was diagnosed with LCA one year after birth based on clinical evaluation due to congenital rotary nystagmus, reduced vision, and strabismus since birth. The patient was the first child of an endogamic couple from the northwest of Spain. The patient also then presented bilateral keratoconus at 12 years of age. In the setting of the corneal ectasia, the patient suffered three episodes of acute corneal hydrops due to disruption of Descemet's membrane. The ophthalmic data were obtained at 27 years old. Visual acuity was reduced to light perception. The central cornea at both eyes has a pronounced cone shape, hydrops, and small leucomas. Keratoconus-associated Munson's sign was also detected on the lower eyelid. Fundus showed vascular attenuation, bone-spicule pigmentation in the mid-periphery and papillar gliosis. The Asper LCA microarray chip identified a previously known homozygous variant NM_014336.4:c.265T>C; p.(Cys89Arg) in AIPL1[1]. Further Sanger sequencing showed correct segregation in both parents.

RNA extraction and Taqman Assay

Total RNAs were extracted from 15-20 ROs per time point using RNeasy kit (Quiagen) and RNA yields and quality were checked with a NanoDrop spectrophotometer (Thermo Fischer Scientific). cDNA was synthesized from 1 µg of mRNA using the QuantiTect reverse transcription kit (Qiagen). qPCR reaction performed on Light Cycler 480, Roche with 20 ng of cDNA and TaqMan® Gene expression Master Mix (Thermo Fischer Scientific) and FAM labelled TaqMan probes (**Supplementary table S2**) following manufacturer's instructions. The PCR cycling program consisted of denaturing at 95°C for 10 min followed by 40 cycles of 95°C for 15 sec and annealing/elongation at 60°C for 1 min. Results were normalized against POL2A and quantification of gene expression was based on the DeltaCt Method in minimum 3 (N>3) differentiation experiments for each genotype. Data from Control 1 and Control 2 were pooled and represented as Control. Statistical analysis was performed using GraphPad Prism 5 with two way ANOVA test with Bonferroni post test. Values of $P < 0.05$ were considered statistically significant.

Strand-specific RNA-seq and data analysis

Retinal organoids (15-20) from Control 1, Control 2 and AIPL1-LCA from 3-5 independent differentiation experiments were collected at indicated time points during the differentiation. Total RNA was extracted using the RNeasy Mini Kit (Qiagen, Hilden, Germany), and treated with DNase I to remove any genomic DNA contamination.

Directional RNA-seq and primary data analysis were performed as previously described[2]. Quality control, sequence alignment, transcript and gene-level quantification of primary RNA-seq data was done via an established bioinformatics pipeline[3, 4] except the whole genome alignment and by excluding non-coding RNAs from target transcriptome. All other bioinformatics analyses were performed in the *R* statistical environment. Genes were kept for further analysis only if there were ≥5 count per millions (CPM) in all replicates of at least one group. The data were subjected to TMM normalization by edgeR v3.10.5, and PCA and Pearson correlation were performed with normalized log2 CPM values. Co-inertia analysis (CIA) was performed with R package called "made4". All the dimension reduction plots are custom. The organoid transcriptome data from the two control lines was comparable by PCA and therefore combined for DE analysis against the patient organoids. DE analysis was performed using limma v3.24.15, and genes having ≥ 2-fold change at least between 2 time points in one of the genotyped transcriptomes and a false discovery rate (FDR) ≤ 0.01 were considered to be significantly differentially expressed. Gene expression clustering was performed on gene-wise Z-scores using Affinity Propagation (AP) (apcluster v1.4.3 package[5] with k-means option where k=12 by choosing corSimMat function as similarity measure. To determine the number k, PCA projections of genes by using their z-scores were used. Since the sample projections shows time as the primary source of variation same would be the true for the gene projections however the number of the genes is incomparably higher than the sample number, one axis would not be enough to explain the time. Therefore, the angles between x-axis and the imaginary vector from origin to each gene projected coordinate were sorted after converting them to radians. Sorted angle transformations then divided to four groups (one per quadrant). Then resulting 4 heatmaps were integrated via eyeballing, and the optimal number of clusters became visible. GO enrichment analysis was performed using clusterProfiler v3.2.14[6]. To reduce redundancy associated with GO analysis, we performed semantic similarity comparisons of the enriched GO terms using GOSemSim v2.0.4[7] and semantic similarities clustered with AP. The term with the highest level in each cluster is taken as the enriched GO term. Heatmaps

of GO term gene lists were generated using the negDistMat order of AP on the log2 CPM values. All the heatmaps are the products of customized ggplots.

SUPPLEMENTAL FIGURES

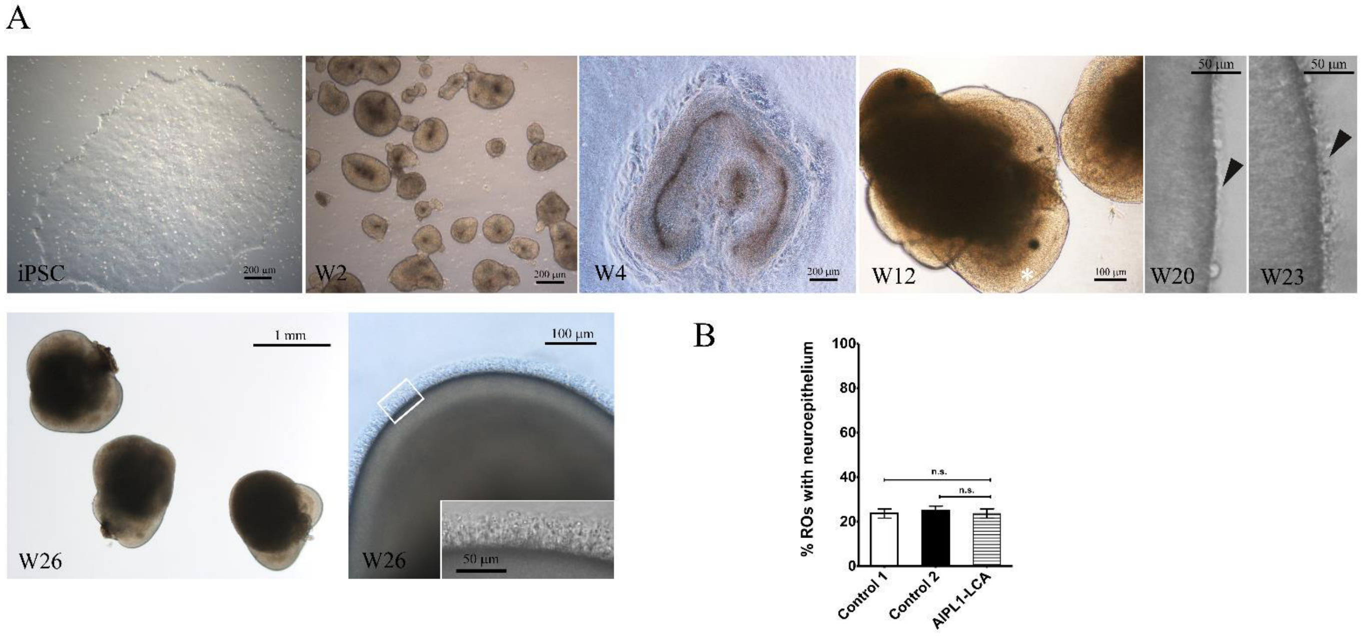

Figure S1. Differentiation of control hiPSC lines toward ROs. A. Phase contrast micrographs of the differentiation stages of Control 1 ROs: hiPSCs, the floating aggregates after treating the hiPSCs with dispase (W2), aggregates plated on Matrigel GFR-coated plates reach a typical morphology by week 4 (W4), are dissected manually and are grown in suspension thereafter. The typical transparent NR domain (*) is formed by W12. At W20 the projections (arrowhead) begin to emerge at the apical edge of organoids. The projections are more abundant at W23. At W26 the organoids have reached 1-1,5 mm in diameter and show dense translucent projections at the apical edge. Inset shows higher magnification of the apical edge with protrusions. B. Efficiency of neuroepithelium formation in ROs. Data were analyzed by One way ANOVA test. The differences between groups were non-significant (n.s.); data are mean ± s.e.m¸ N=3 independent experiments with >288 ROs each.

Abbreviations: RO, retinal organoids; NR, neural retina; W, week; GFR, growth factor reduced.

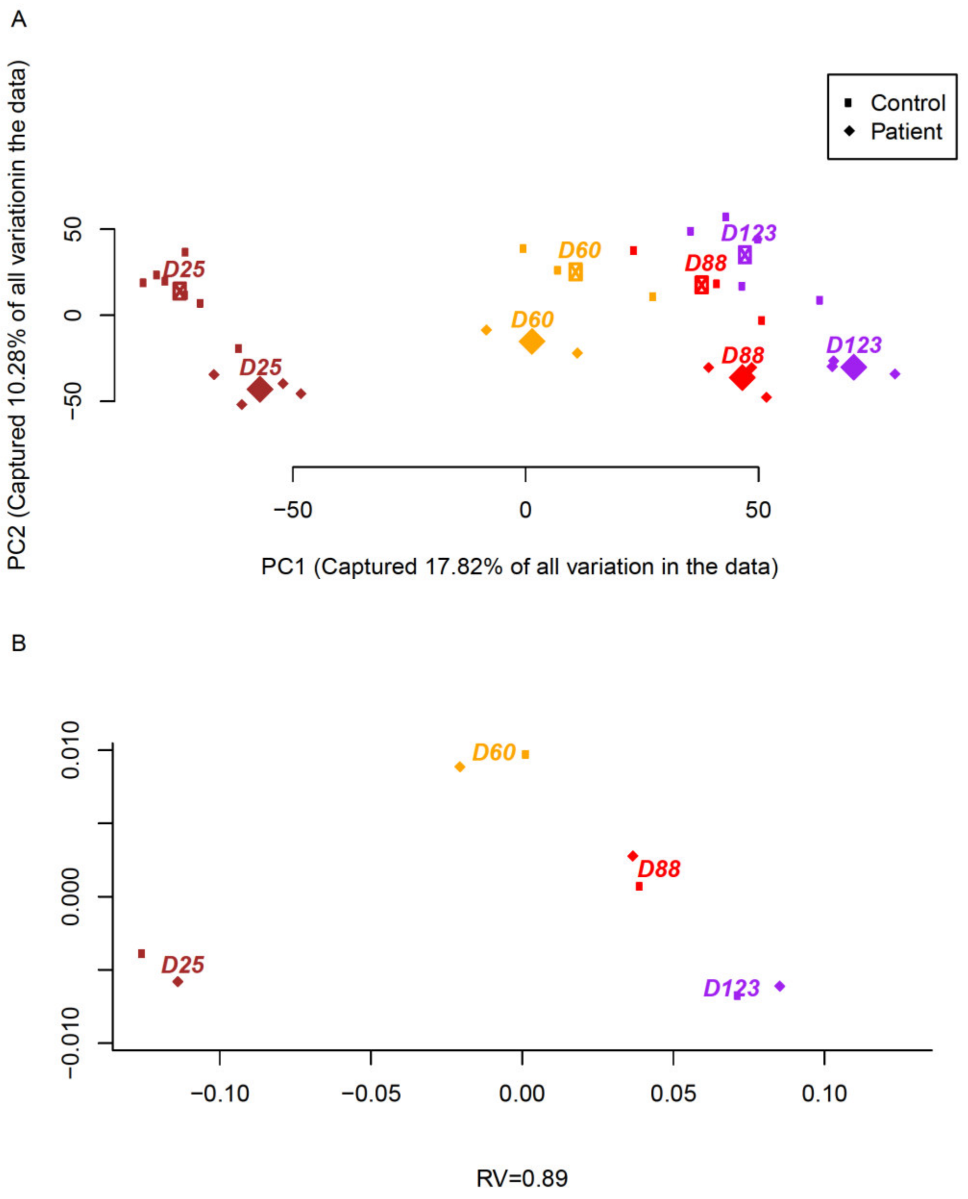

Figure S2. Projection of the raw data into two dimensions: A-) PCA and B-) Time match with CIA analysis. RV coefficient is a measure of time match between two datasets in a range between 0 to 1 where 0 means no relationship and 1 perfect match. In PCA analysis all the samples were used (small circle and diamonds). For CIA mean expression of each gene within samples belonging to same time point were taken in order to simplify the comparison. The labels were written to the mean of coordinates of sample groups in both.

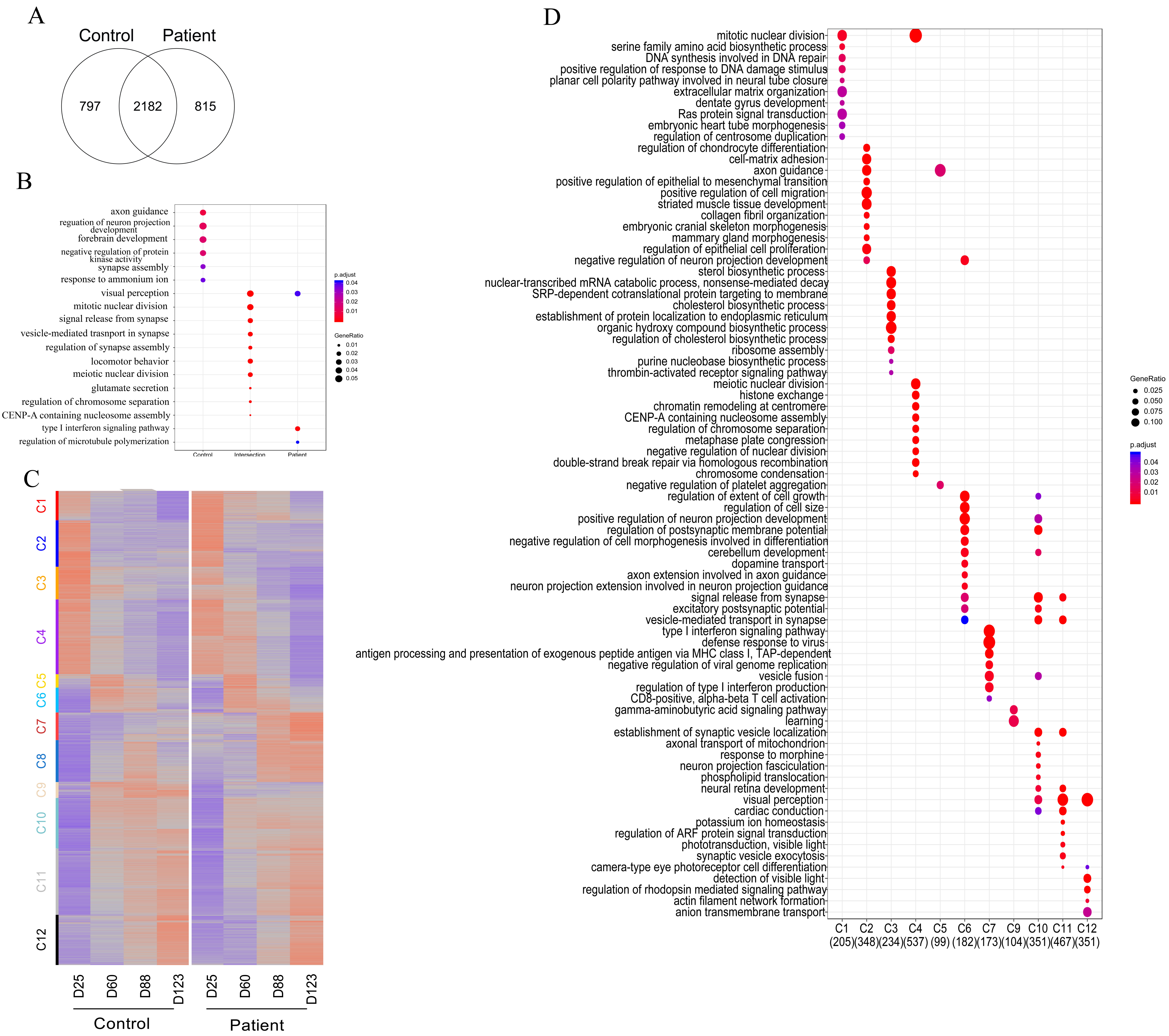

A
Control
Patient
797
2182
815
B
axon guidance
regulation of neuron projection development
forebrain development
negative regulation of protein kinase activity
synapse assembly
response to ammonium ion
visual perception
mitotic nuclear division
signal release from synapse
vesicle-mediated trasnport in synapse
regulation of synapse assembly
locomotor behavior
meiotic nuclear division
glutamate secretion
regulation of chromosome separation
CENP-A containing nucleosome assembly
type I interferon signaling pathway
regulation of microtubule polymerization
Control
Intersection
Patient
p.adjust
0.04
0.03
0.02
0.01
GeneRatio
0.01
0.02
0.03
0.04
0.05
C
C1
C2
C3
C4
C5
C6
C7
C8
C9
C10
C11
C12
D25
D60
D88
D123
D25
D60
D88
D123
Control
Patient
D
mitotic nuclear division
serine family amino acid biosynthetic process
DNA synthesis involved in DNA repair
positive regulation of response to DNA damage stimulus
planar cell polarity pathway involved in neural tube closure
extracellular matrix organization
dentate gyrus development
Ras protein signal transduction
embryonic heart tube morphogenesis
regulation of centrosome duplication
regulation of chondrocyte differentiation
cell-matrix adhesion
axon guidance
positive regulation of epithelial to mesenchymal transition
positive regulation of cell migration
striated muscle tissue development
collagen fibril organization
embryonic cranial skeleton morphogenesis
mammary gland morphogenesis
regulation of epithelial cell proliferation
negative regulation of neuron projection development
sterol biosynthetic process
nuclear-transcribed mRNA catabolic process, nonsense-mediated decay
SRP-dependent cotranslational protein targeting to membrane
cholesterol biosynthetic process
establishment of protein localization to endoplasmic reticulum
organic hydroxy compound biosynthetic process
regulation of cholesterol biosynthetic process
ribosome assembly
purine nucleobase biosynthetic process
thrombin-activated receptor signaling pathway
meiotic nuclear division
histone exchange
chromatin remodeling at centromere
CENP-A containing nucleosome assembly
regulation of chromosome separation
metaphase plate congression
negative regulation of nuclear division
double-strand break repair via homologous recombination
chromosome condensation
negative regulation of platelet aggregation
regulation of extent of cell growth
regulation of cell size
positive regulation of neuron projection development
regulation of postsynaptic membrane potential
negative regulation of cell morphogenesis involved in differentiation
cerebellum development
dopamine transport
axon extension involved in axon guidance
neuron projection extension involved in neuron projection guidance
signal release from synapse
excitatory postsynaptic potential
vesicle-mediated transport in synapse
type I interferon signaling pathway
defense response to virus
antigen processing and presentation of exogenous peptide antigen via MHC class I, TAP-dependent
negative regulation of viral genome replication
vesicle fusion
regulation of type I interferon production
CD8-positive, alpha-beta T cell activation
gamma-aminobutyric acid signaling pathway
learning
establishment of synaptic vesicle localization
axonal transport of mitochondrion
response to morphine
neuron projection fasciculation
phospholipid translocation
neural retina development
visual perception
cardiac conduction
potassium ion homeostasis
regulation of ARF protein signal transduction
phototransduction, visible light
synaptic vesicle exocytosis
camera-type eye photoreceptor cell differentiation
detection of visible light
regulation of rhodopsin mediated signaling pathway
actin filament network formation
anion transmembrane transport
C1 (205)
C2 (348)
C3 (234)
C4 (537)
C5 (99)
C6 (182)
C7 (173)
C9 (104)
C10 (351)
C11 (467)
C12 (351)
GeneRatio
0.025
0.050
0.075
0.100
p.adjust
0.04
0.03
0.02
0.01

Figure S3. DE analysis of RNA-Seq data with respect to time in both transcriptomic datasets. A-) the Venn diagram showing the distribution of number of time-responsive genes across the genotype. B-) GO term (Biological Process) enrichment analysis of the genes categorized in the Venn. C-) sorted and clustered (K-Means where k=12) heatmap of all DE genes with respect to time change. D-) GO term (Biological Process) enrichment analysis of the genes categorized (12 clusters) in the heatmap. Some clusters did not show any enrichment.

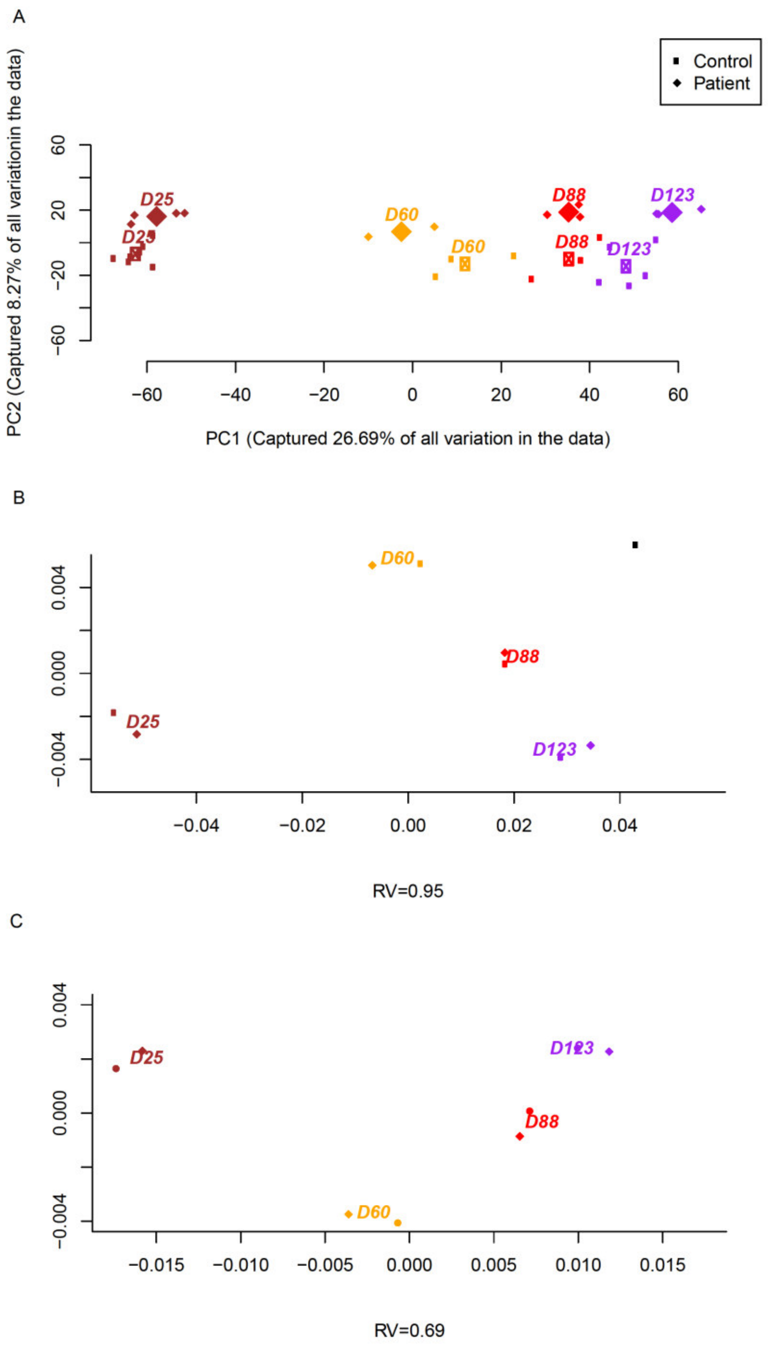

Figure S4. Projection of the data into two dimensions with DE genes: A-) PCA and B-) Time match with CIA analysis with intersected genes. C-) Time match with CIA analysis with unique DE genes. In PCA analysis all the samples were used (small circle and diamonds). In CIA samples belonging to same time point were meaned gene-wise. The labels were written to the mean of coordinates of sample groups in all three.

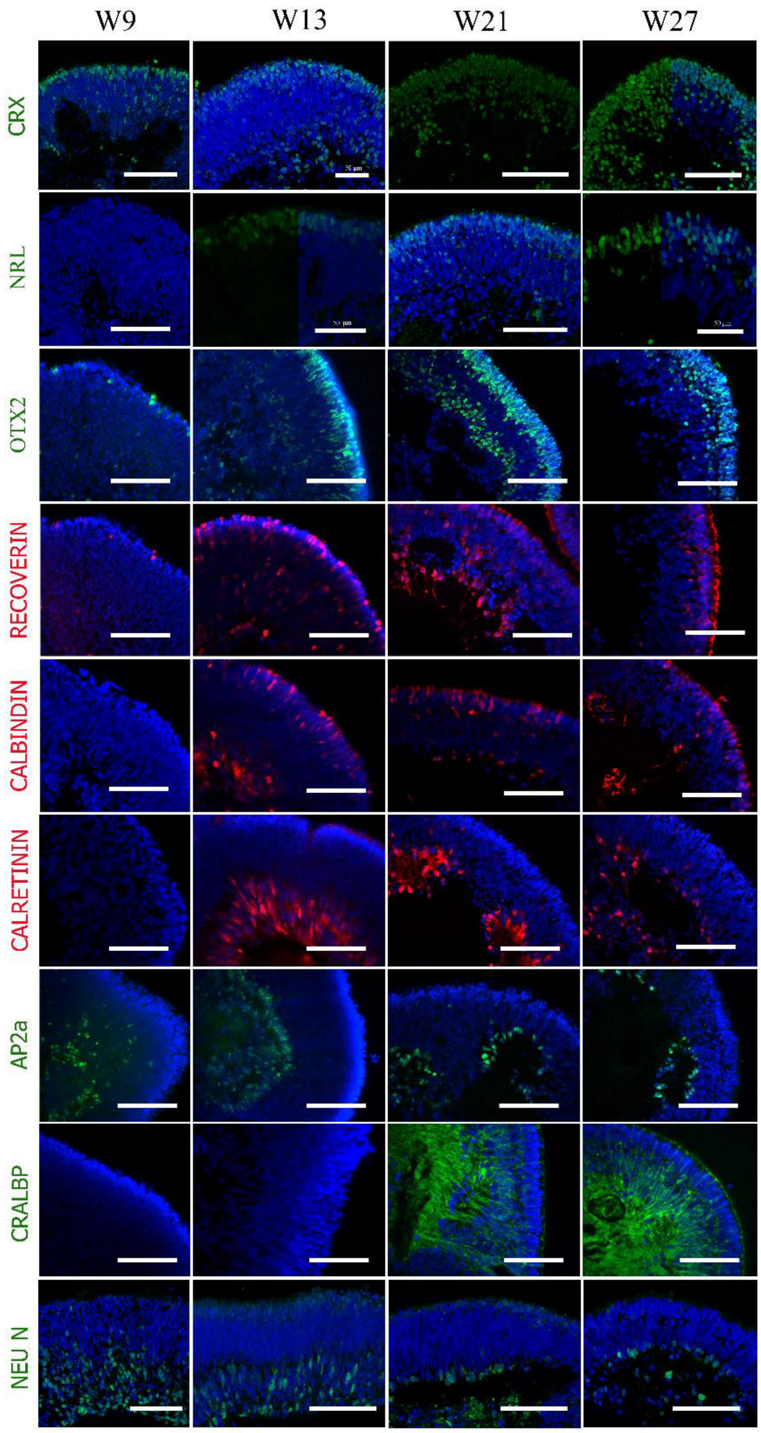

Figure S5. Time course of retinal cell marker expression during the differentiation of AIPL1-LCA organoids. Photoreceptor marker expression: OTX2, CRX, NRL, RECOVERIN. Apical accumulation can be observed for all photoreceptor markers. CALBINDIN stains horizontal cells and cone cells. Amacrine cells are detected by CALRETININ and AP2. CRALBP immunolabels Müller cells that extend across the entire ROs. NEUN staining of ganglion cells. Nuclei were counterstained with 4′,6-diamidino-2-phenylindole (DAPI, blue); panels CRX W27, NRL W13 and NRL W27 show nuclei counterstained with DAPI on the right half of the image. Scale bar 100 µm except where indicated differently.

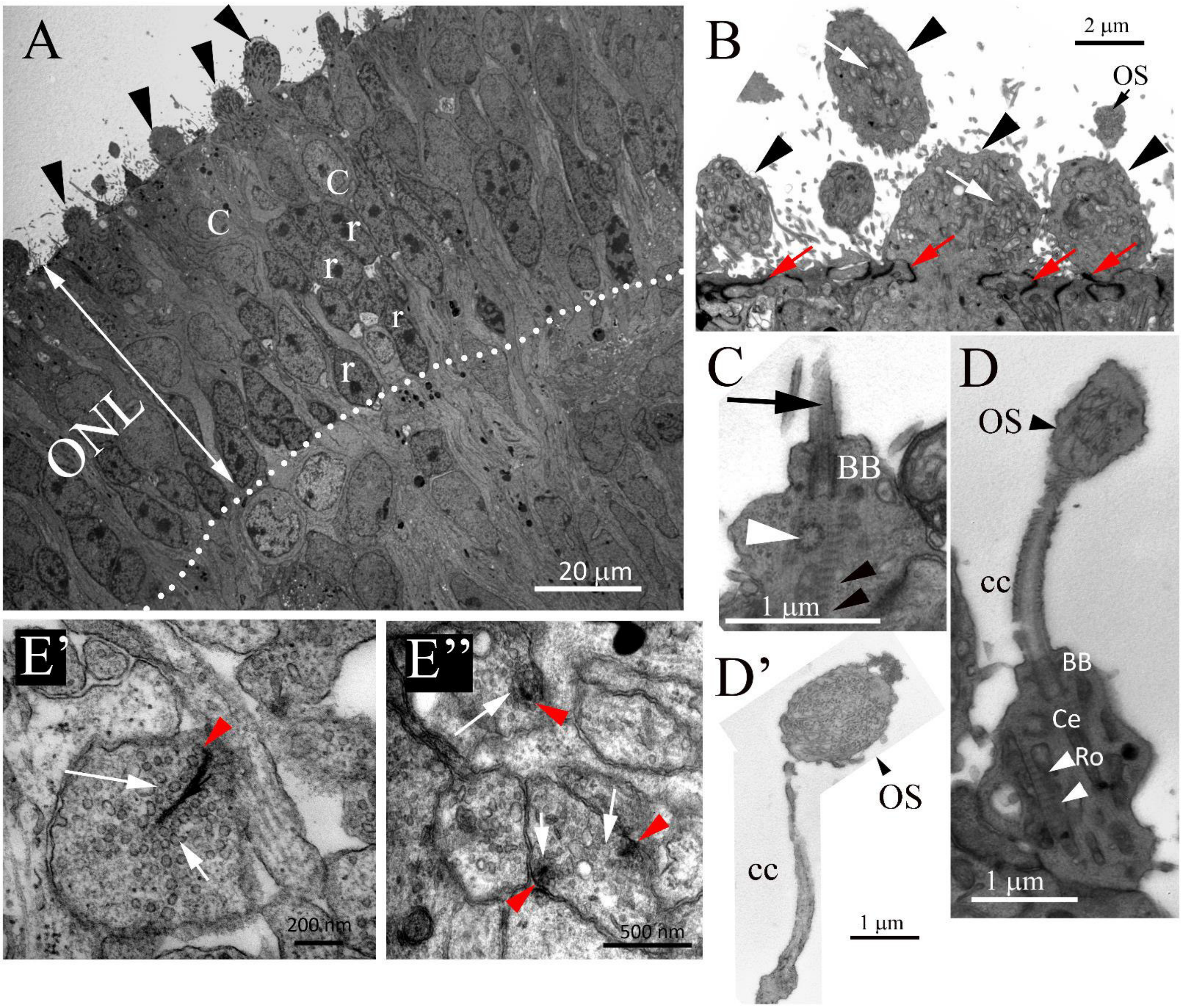

Figure S6. Representative electron micrographs of Control 1 ROs at W27. A. Ultrastructure of the ONL-like region: ISs protruding at the apical side of the organoid (black arrowheads), cone-like cells (C) at the outermost side of the ONL with large cell body and large bright nucleus and deeper aligned, presumably rods with dark oval nuclei (r). Dotted line: the basal edge of ONL, synaptic ribbons were identified underneath (E and E').

B. ISs (black arrowheads) at the apical border with elongated mitochondria (white arrow) and OLM (red arrows). Presumptive rudimentary OS (black arrow).

C. Longitudinal section of CC (black arrow) arising from the IS with the BB at the base. Dark cross-striation representing rootlet filaments protruding from the BB (black arrowheads). The transversal section of the CC (white arrowhead).

D. CC arising from the IS protruding into the organelle free vesicles, presumably presenting nascent OS. Black arrowhead pointing toward membranous disk precursors.

D'. Another example of CC protruding into the presumptive OS vesicle with developing membrane disc stacks.

E' and E''. Higher magnification of the inset in A, beyond dark rod nuclei, showing multiple synaptic ribbons with electron dense bars (red arrowheads) surrounded by presynaptic vesicles (black arrows).

Abbreviations: ONL, outer nuclear layer; ISs, inner segments; OLM, outer limiting membrane; CC, connecting cilium; BB, basal body; Ce, centriole. OS, outer segment, Ro, Rootlet.

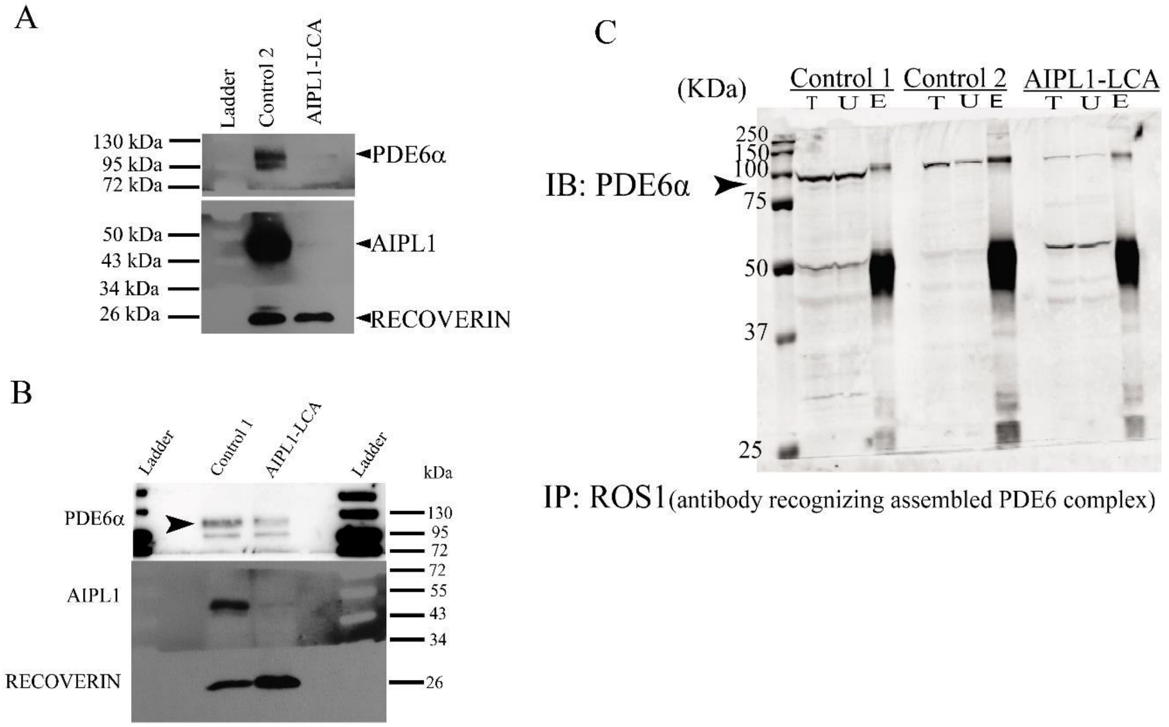

Figure S7. Supporting western blot images for Figure 5D and 5E**.** A**.** Western blotting of AIPL1 (MW 43 kDa) and PDE6α (MW 99 kDa) expression in 27-week ROs. Photoreceptor-specific protein Recoverin (MW 26 kDa) was used as internal control. B. Full western blot images for **Figure 5D**. C. Immunoprecipitation with ROS-1 monoclonal antibody followed by immunoblotting with PDE6α rabbit antibody. PDE6α (MW 99 kDa) expression is shown as Total protein fraction (T), unbound fraction (U), Elution fraction (E).

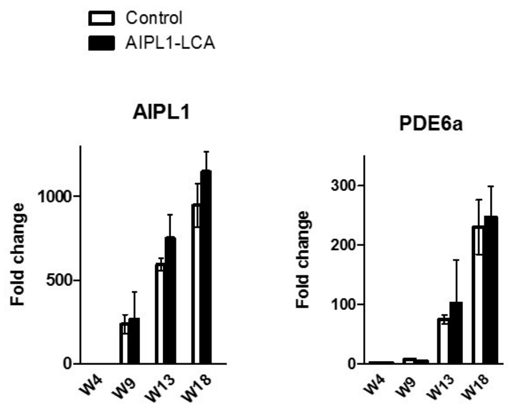

Figure S8. RT-qPCR analysis of *AIPL1* and *PDE6a* expression in retinal organoids at W9, W13 and W18. Data are normalized to retinal organoids at W4. Values plotted as mean ± SEM: Two-way ANOVA was used to compare means. The differences between AIPL1-LCA and control retinal organoids were non-significant.

Supplementary table S1. List of used antibodies

Antibody	Species	Dilution	Reference
**TUJ1**	Mouse	1:500	Neuromics MO15013
**CRALBP**	Mouse	1:250	Abcam15051
**CALBINDIN**	Rabbit	1:500	SWANT CB38
**CALRETININ**	Rabbit	1:500	SWANT CR7697
**AIPL1**	Rabbit	1:10.000	Policlonal antiserum affinity purified; V.Ramamurthy, UWV
**Rhodopsin**	Mouse	1:1000	Millipore MAB5356
**AP2a**	Mouse	1:35	DSHB (3B5)
**PDE6α**	Rabbit	1:1000	Abcam ab5659
**PDE6α**	Rabbit	IB	Invitrogen Prod# PA1-720
**ROS-1**	Mouse	IB	Dr. Joe Beavo (University of Washington)
**OTX2**	Goat	1:20	R&D AF1979
**RECOVERIN**	Rabbit	1:200	Millipore AB5585
**Arl13b**	Rabbit	1:200	Proteintech 17711-1-AP
**Anti-Transducin**	Rabbit	1:300	Tralpha1A (aa 85-103, from M. Simon)*
**NeuN**	Mouse	1:500	Millipore MAB377
**OPSIN LM**	Rabbit	1:500	Millipore AB5405
**OPSIN S**	Rabbit	1:500	Millipore AB5407
**GFAP**	Rabbit	1:400	DAKO Z0334
**NRL**	Goat	1:200	RD AF2945
**ARRESTIN3**	Rabbit	1:1000	Novus NBP2-41249
**Rod ARRESTIN**	Mouse	1:300	against the C10C10 epitope of arrestin; Gift A.Méndez; Univ. of Barcelona
**CRX**	Mouse	1:100	Abnova H00001406-MO2

Phototransduction in transgenic mice after targeted deletion of the rod transducin α-subunit P. D. Calvert, N. V. Krasnoperova, A. L. Lyubarsky, T. Isayama, M. Nicoló, B. Kosaras, G. Wong, K. S. Gannon, R. F. Margolskee, R. L. Sidman, E. N. Pugh Jr., C. L. Makino, and J. Lem PNAS December 5, 2000 97 (25) 13913-13918;

Supplementary table S2. List of TaqMan® Gene Expression ID Assays used for qRT-PCR

Gene symbols	Assay ID
POL2A	Hs00172187_m1
AIPL1	Hs00204945_m1
PDE6a	Hs00166495_m1

REFERENCES

1. Jacobson SG, Cideciyan AV, Aleman TS, et al. Human retinal disease from AIPL1 gene mutations: foveal cone loss with minimal macular photoreceptors and rod function remaining. Investigative ophthalmology & visual science. 2011;52:70-79.
2. Brooks MJ, Rajasimha HK, Roger JE, et al. Next-generation sequencing facilitates quantitative analysis of wild-type and Nrl(-/-) retinal transcriptomes. Molecular vision. 2011;17:3034-3054.
3. Chen HY, Kaya KD, Dong L, et al. Three-dimensional retinal organoids from mouse pluripotent stem cells mimic in vivo development with enhanced stratification and rod photoreceptor differentiation. Molecular vision. 2016;22:1077-1094.
4. Hoshino A, Ratnapriya R, Brooks MJ, et al. Molecular Anatomy of the Developing Human Retina. Developmental cell. 2017;43:763-779 e764.
5. Bodenhofer U, Kothmeier A, Hochreiter S. APCluster: an R package for affinity propagation clustering. Bioinformatics. 2011;27:2463-2464.
6. Yu G, Wang LG, Han Y, et al. clusterProfiler: an R package for comparing biological themes among gene clusters. Omics : a journal of integrative biology. 2012;16:284-287.
7. Yu G, Li F, Qin Y, et al. GOSemSim: an R package for measuring semantic similarity among GO terms and gene products. Bioinformatics. 2010;26:976-978.
